# A new name and a new synonym in *Miconia* (Melastomataceae)

**DOI:** 10.3897/phytokeys.3.1174

**Published:** 2011-05-30

**Authors:** Susanne S. Renner, Renato Goldenberg

**Affiliations:** 1 Herbaria and University of Munich, Menzingerstr. 67, Munich, Germany; 2 Departamento de Botânica, Universidade Federal do Paraná, 81531-970, Curitiba, Paraná, Brazil

**Keywords:** Bolivia, Colombia, Melastomataceae, *Miconia*, homonyms, synonyms, taxonomy

## Abstract

The name *Miconia densiflora* Cogn. (1886) is a later homonym of *Miconia densiflora* (Gardner) Naudin (1851), but since we propose it as a taxonomic synonym of *Miconia caudata* (Bonpl.) DC. (1828), we do not provide a new name. The name *Miconia longicuspis* Herzog (1909) is a later homonym of *Miconia longicuspis* Cogn. (1891) and we here propose its replacement by *Miconia longicuspidata* S.S. Renner & R. Goldenb.

## Introduction

The site Melastomataceae.net provides a portal to open-access databases dealing with Melastomataceae, a pan-tropical family of about 3500 species. Among the databases that can be accessed at this site is “MEL names,” which deals with the ca. 13,278 names of Melastomataceae and Memecylaceae (= Melastomataceae subfamily Olisbeoideae) and provides information on recognized species, synonyms, and relevant literature. In the course of dealing with the 1497 names available for *Miconia* Ruiz & Pav., a genus of at least 1061 accepted species, we discovered two homonymy problems, resolved here. Since one of the homonyms is actually a heterotypic synonym of another species, a replacement name is only needed for one of these species. A comprehensive taxonomic treatment of *Miconia* is currently in preparation, as part of the PBI Miconieae project (sweetgum.nybg.org/melastomataceae/ ).

## Systematics

### Miconia longicuspidata

S.S.Renner & R.Goldenb. nom. nov.

urn:lsid:ipni.org:names:77111574-1

http://species-id.net/wiki/Miconia_longicuspidata

Miconia longicuspis  Herzog, Feddes Repert. Nov. Sp. 7: 64. 1909. Type: Bolivia. Cordillera de Santa Cruz: Cerro Amboró, Cuñucú, 600-1400 m, Oct 1907, T.Herzog 326 (holotype: Z!). Not *Miconia longicuspis* Cogn. in A.DC. & C.DC, Monog. Phan. 7: 850. 1891. (Replaced name)

#### Comments.

*Miconia longicuspis* Herzog is a later homonym of *Miconia longicuspis* Cogn., a treelet from eastern Brazil (Goldenberg and Reginato 2006). *Miconia longicuspidata* is known only from the holotype at Z, a collection by Theodor Herzog (1880-1961) in the mountains of Cuñucú, Bolivia, in 1907. We have found no isotypes or type photos in G, JE, L, U, W, or WAG. This species most closely matches *Miconia abbreviata* Markgr., a widespread shrub that ranges from Bolivia throughout the Amazon basin to the Guianas, but differs in the much denser secondary venation. In *Miconia abbreviata* the secondaries are spaced at about 0.5–0.7 mm apart, in *Miconia longicuspidata*, only 2–3 mm apart. Theodor Herzog was an expert mountaineer and collector, who explored widely in Bolivia. During his two expeditions to that country in 1907/08 and 1910/11, he collected about 600 species of vascular plants. In 1910, Herzog not only explored the Cordillera Santa Cruz, where *Miconia longicuspidata* appears to be endemic, but also surveyed the Cordillera de Cocapata, a range of peaks near Cochabamba extending northwest for about 100 kilometers. A description of the flora of the Cordillera de Santa Cruz is given in Herzog (1910). A color photograph of the type is available at http://www.zuerich-herbarien.uzh.ch.

### Miconia caudata

(Bonpl.) DC., Prodr. 3: 187. 1828.

http://species-id.net/wiki/Miconia_caudata

Melastoma caudata  Bonpl., Monogr. Melast. 1: 13. t. 7. 1807.(Basionym)
Miconia densiflora  Cogn., Bot. Jahrb. 8(1): 22, 1887 [1886]; et in A. DC. & C. DC. Monog. Phan. 7: 744, 1891, *syn. nov*. Type: COLOMBIA. Cauca: Popayan, Apr 1883, Lehmann 2798 (Isotype: US!). Non *Miconia densiflora* (Gardner) Naudin, Ann. Sc. Nat. Ser. 3, 16: 245, 1851, basionym *Chaenopleura densiflora* Gardner, Hook. Lond. Journ. Bot. 2: 349, 1843. (New synonym)

#### Comments.

*Miconia densiflora* Cogn. is a later homonym of *Miconia densiflora* (Gardner) Naudin, which in turn is a taxonomic synonym of *Miconia pusilliflora* (DC.) Triana, a shrub from eastern Brazil (Cogniaux 1891; Goldenberg 2000). By contrast, Cogniaux’ *Miconia densiflora* is based on a collection made by Lehmann near Popayán, in the State of Cauca, Colombia, in 1883. The name is undoubtedly a taxonomic synonym of the common and frequently collected *Miconia caudata* (Bonpl.) DC. Cogniaux (1891: 736, 739) erroneously states that *Miconia caudata* has glandular-pubescent filaments, while *Miconia densiflora* has glabrous ones. However, all 15 specimens of *Miconia caudata* that we checked had completely glabrous filaments. A color photograph of *Miconia densiflora*’s type is available at http://www.botany.si.edu/types.

## Supplementary Material

XML Treatment for Miconia longicuspidata

XML Treatment for Miconia caudata
